# Quantification of adsorbed and dangling citrate ions on gold nanoparticle surface using thermogravimetric analysis

**DOI:** 10.1038/s41598-020-65013-0

**Published:** 2020-05-19

**Authors:** Manish Bajaj, Nishima Wangoo, D. V. S. Jain, Rohit K. Sharma

**Affiliations:** 1 Department of Chemistry and Centre for Advanced Studies in Chemistry, Panjab University, Chandigarh, 160014 India; 2 Department of Applied Sciences, University Institute of Engineering and Technology, Panjab University, Sector 25, Chandigarh, 160014 India

**Keywords:** Soft materials, Soft materials, Nanoparticles, Nanoparticles

## Abstract

A novel approach involving thermo-gravimetricanalysis (TGA) for the quantification of citrate ions present on the surface of gold nanoparticles has been reported. TGA study was carried out on AuNPs in response to parameters such as concentration of tri-sodium citrate and pH of gold nanoparticles depicting that the number of citrate ion present on gold nanoparticles is highly pH dependent. In general, the citrate ions were observed to be higher in alkaline conditions contradicting earlier beliefs. These results also underline the significance of TGA as a novel tool for quantification of citrate molecules present on gold nanoparticle surface. Thus, the present approach not only provides with an insight into mechanistic details of gold nanoparticle synthesis but also opens the usage of TGA for understanding the nano range association of molecules.

## Introduction

Gold nanoparticles (AuNPs) have gained considerable popularity as one of the most stable metal nanoparticles with unique electrical, optical and surface chemical properties making them applicable in fields such as sensing, drug delivery, cancer therapy and tissue engineering^1–4^. It is well known that AuNPs can be synthesized using different chemical reduction methods; out of which the citrate method introduced by Turkevich is the most widely used for the preparation of AuNPs^5,6^ The biocompatible nature of citrate ion and its ability to act as a cross-linker between various ligands along with its labile nature with respect to displacement by various ligands on the surface of AuNPs render Turkevich method as one of the most popular for AuNPs synthesis^7,8^. However, in spite of the popularity and extensive usage of citrate based methods for synthesis of AuNPs^9,10^, there has been limited introspection in literature into the probable interaction between citrate and gold ions along with the structural details of citrate adsorption.

The mechanism of adsorption of citrate ions on gold surface reported so far discuss this issue more from qualitative perspective than quantitative^11,12^ In an earlier such report, the structural details of adsorption of citrate ion have been discussed exhibiting the formation of three carboxylate groups coordinated as η-2 bonded carboxylate adsorbate with characteristic infrared absorbance at around 1390 cm^-1^ coordinated to the Au (111) surface in an acidic electrolyte^13^. Similarly, subtractively normalized interfacial Fourier transform infrared spectroscopy has been used for quantification of surface coverage of AuNPs (~3 × 10^-10^ mol/cm^2^). This is based on the fact that all three carboxylic groups of citric acid undergo deprotonation^14^.The authors have also postulated that the carboxylate groups take a tilted conformation on AuNP surface. Further, the thermodynamic description of adsorption of citric acid and dihydrogen citrate on Au (111) surface has been studied using electrochemical methods such as chronocoulometry and cyclic voltammetry comparing the adsorption of citrate at different pH^15^. The general observation was that the surface coverage increased three-fold as pH was raised from 1 to 3.In a recent study, the effect of thedecrease in pH from 5.3 to 4.7 resulted in the reduction of the AuNP concentrationby 46%^16^.

Atomistic molecular dynamics simulations (MD) have been used to understand different binding modes of citrate ion onto AuNP surface where it was concluded that the orientation of citrate is such that carboxylate groups point towards water (solvent) molecules and are stabilized by complex interplay between citrate-gold and citrate-citrate interactions^17^. It is pertinent to mention here that these simulation studies have been based on the assumption that there were only two binding possibilities of citrate ion onto AuNP surface, one through central carboxylate group of citrate alone and other with the terminal carboxylate groups. Similarly, MD based on a reactive force field has been used to investigate the absorption modes of citrate on AuNPs^18^. It was revealed that 50% of the adsorbates binds with AuNPs through one of the terminal carboxyl group while other 50% through both the central and a terminus carboxyl group. In another report using MDS, it has been reported that the dihydrogen citrate is the predominant configuration of citratepresent over AuNP surface^19^.

Recently, binding modes of various carboxylate-containing systems such as citrate, acetate, succinate and glutarate with AuNPs has been examined using ^13^C and ^23^Na solid-state NMR^20^. It has been reported that these systems possessed three different modes to AuNPs surface; out of which monodentate mode is most favoured. Further, the conformation of citrate anions on AuNPs was investigated using IR and X-ray photoelectron spectroscopy which basically discussed the adsorption of citrate anions through the central carboxylate group^21^. This was supported by structure-based model of citrate adsorption on Au(111) in which only syn conformers i.e. mono-sodium citrate (MSC) was adsorbed in bridge sites which interacted with each other and dangling citrate. The alkaline conditions convert the monodentate citrate ion into tri-sodium citrate (TSC) which undergoes tetradentatecoordination upon binding of the hydroxyl and carboxyl groups to the surface. Based on these findings, it was proposed that the formation of citrate layer is favoured on Au(111) but deterred over Au(110) and Au(100). However, no experimental evidence was provided and this as well as earlier discussed reports did not focus on quantification of citrate ions over nanoparticle surface.

**Figure 1:**
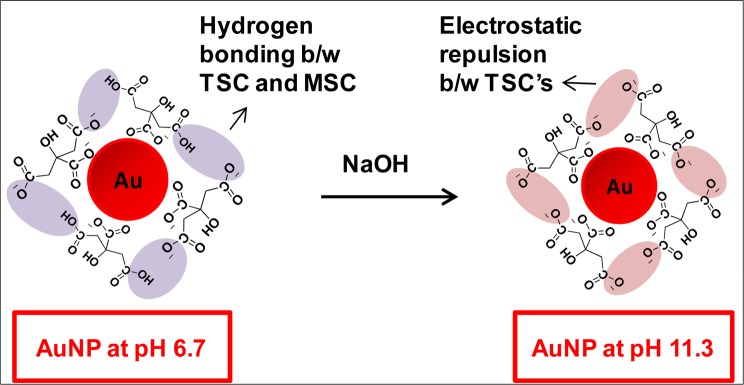
Schematic reaction illustrating different binding of citrate ions on the surface of AuNPs (at pH 6.7 and pH 11.3).

Keeping the above considerations in mind, the present report is a major step forward in understanding the actual mechanistic behaviour of citrate ions on Au surface. Herein, the concentration of citrate ions present on AuNPs have been tried to be quantified using thermogravimetric analysis (TGA) carried out on the dried AuNPs synthesized by Turkevich method (Fig. 1). The behaviour of citrate ions on nanoparticle surface was further assessed by varying reaction parameters such as concentration of TSC and pH of the colloidal solution. The results presented here are quite significant as we have found that surface concentration increases as the pH is raised contrary to most of the earlier studies^21^. These findings also underline the importance of using TGA as a new tool for quantification of citrate anions onto metallic nanoparticle surfaces. It is pertinent to mention here that to the best of our knowledge, this is the first report where the mechanism of AuNPs synthesis using Turkevich has been tried to be understood based on actual quantification of citrate ions.

Initially, the concentration of TSC was varied from 34 mM to 68 mM to synthesize AuNPs using Turkevich method. It was observed that the increase in the concentration of the TSC led to a blue shift in the absorption maxima which may be attributed to the size variation of AuNPs (Fig. S1). Also, it is known that the ratio of gold ion to TSC concentration have a significant effect on the morphology of the citrate-stabilized AuNPs^22^. Further to check it, TEM analysis was performed which showed the spherical shape of AuNPs with monodispersive nature and the average size of the AuNPs was found to be of the order of 18 (±1) nm (termed as Au-18) and 15 (±1) nm (termed as Au-15) (calculated from the histograms obtained with the help of ImageJ software) corresponding to 34 mM and 68 mM citrate concentrations, respectively (Fig. S2A–D).

**Figure 2:**
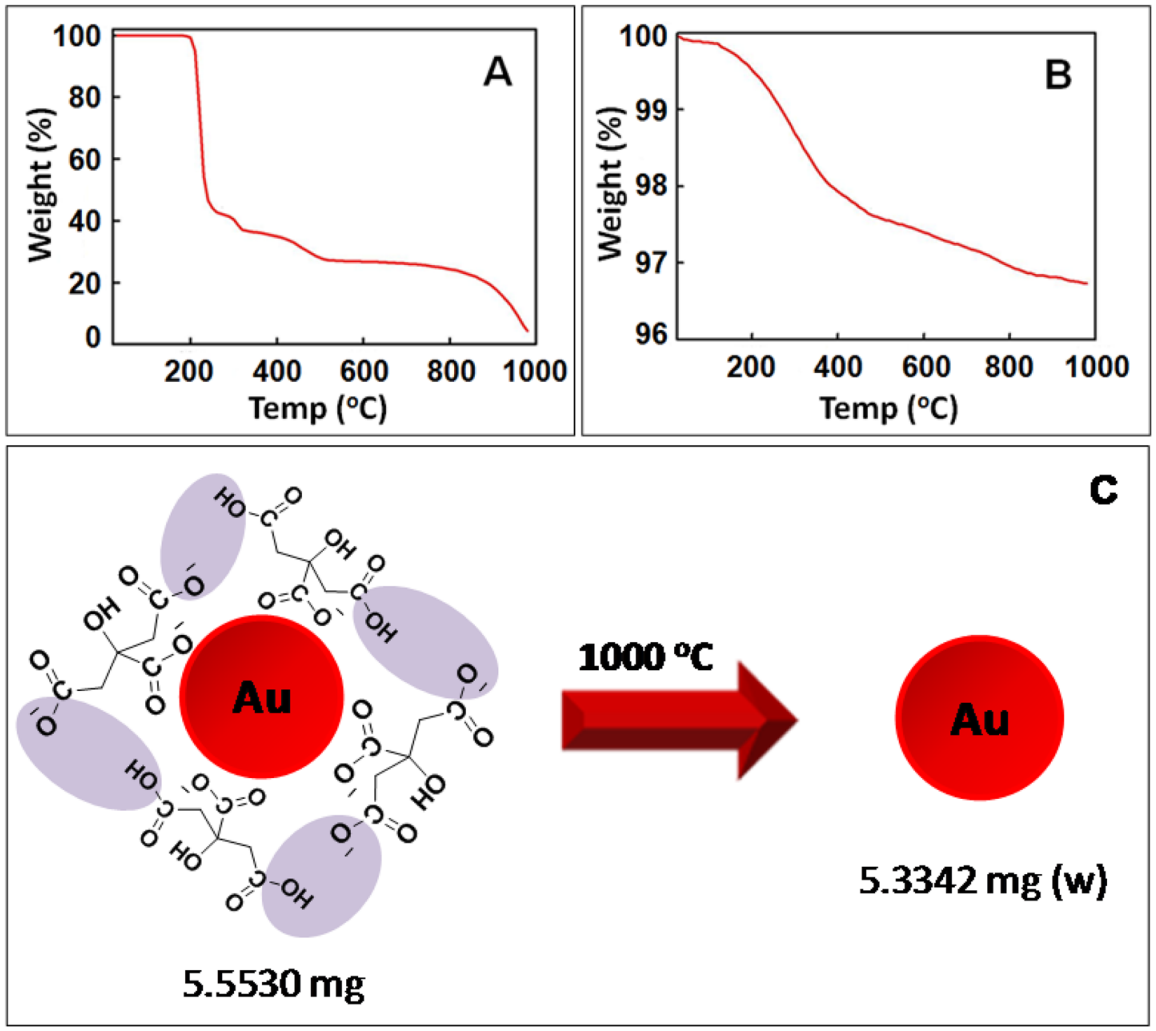
TG curve (Batch I) (**A**) MSC, (**B**) Au-18 at pH 6.7; and (**C**) Schematic reaction showing decomposition of Au-18 (at pH 6.7).

As per experimental evidences, it has reported that there exist mainly two types of citrate conformers on the surface of AuNPs during Turkevich synthesis^20,21^. Under neutral pH range, the citrate ions are present mostly in the form of dihydrogen citrate (or MSC) whereas they get converted to TSC in alkaline condition. Keeping this into consideration, the decomposition behaviour of MSC and Au-18 (at pH 6.7) was compared using TGA (Fig. 2A,B). The MSC decomposed completely when heated up to 1000 °C with final mass becoming zero residual mass (Fig. 2A)^23^. On similar lines, in Batch I when Au-18 at pH 6.7 (5.5530 mg) were thermo-gravimetrically analyzed up to same temperature limit (Fig. 2B), the entire MSC decomposed leading to pure gold in form of nanoparticles as left over final product as 5.3342 mg (w) (Fig. 2C). The residual mass of Au and the size of AuNPs as measured by electron microscopy were used to calculate number of AuNPs. In order to find out the actual concentration of citrate ions on the surface of AuNPs, following mathematical equations (eqs.) (1–5) have been used.

1 $${{\bf{V}}}_{{\bf{A}}{\bf{u}}({\bf{l}}{\bf{e}}{\bf{f}}{\bf{t}})}={\rm{w}}/{\rho }_{{\rm{Au}}}$$

2 $${{\bf{N}}}_{{\bf{A}}{\bf{u}}{\bf{N}}{\bf{P}}{\bf{s}}}={{\rm{V}}}_{{\rm{Au}}({\rm{left}})}/{{\rm{V}}}_{{\rm{Au}}}$$

3 $${{\bf{N}}}_{{\bf{t}}}={{\rm{m}}}_{{\rm{m}}}\times {{\rm{N}}}_{{\rm{o}}}$$

4 $${{\bf{N}}}_{{\bf{t}}/{\bf{A}}{\bf{u}}{\bf{N}}{\bf{P}}}={{\rm{N}}}_{{\rm{t}}}/{{\rm{N}}}_{{\rm{AuNPs}}}$$

5 $${{\bf{C}}}_{{\bf{t}}}={{\rm{N}}}_{{\rm{t}}/{\rm{AuNP}}}/{{\rm{A}}}_{{\rm{Au}}}\times {{\rm{N}}}_{{\rm{o}}}$$

In Eq. 1, the amount of Au left (w) after decomposition is divided by density of gold (ρ_Au_) to give the volume of Au left (V_Au (left)_); from which number of AuNPs (N_AuNPs_) can be deduce in Eq. 2 using volume of Au (V_Au_). After that, by multiplying the number of moles of citrate ions lost during decomposition (m_m_) with Avogadro’s number (N_o_) provides the total number of citrate molecules (N_t_) attached to AuNPs (Eq. 3). Further N_t_ and N_AuNPs_ are used to figure out the number of citrate molecules per AuNP (N_t/AuNP_) (Eq. 4). Finally, Eq. 5 will be used to find out the citrate ion concentration (C_t_) covering AuNP surface using N_t/AuNP_, surface area of Au (A_Au_) and N_o_.

Following the above mentioned mathematical Eqs. 1–5 for Au-18 (at pH 6.7), V_Au (left)_ was calculated as 27.63 × 10^−11^ m^3^ (Eq. 1); from which N_AuNPs_ was found to be 9.05 × 10^13^ using V_Au_ (4πr^3^/3 = 3.052 × 10^−24^ m^3^) (Eq. 2). After that, N_t_ was deduced to be 0.504 × 10^18^ (Eq. 3) which further helped to calculate N_t/AuNP_ as 5.569 × 10^3^ (Eq. 4). The A_Au_ (4πr^2^ = 10.17 × 10^−12^ cm^2^) was used to find out C_t_ which is coming out to be 9.09 × 10^−10^ mol/cm^2^ (Eq. 5).

As discussed earlier, under alkaline condition MSC ions convert into the TSC ions on AuNP surface. So, the varying pH has been known to be a crucial factor^16^ affecting the concentration of citrate ions on AuNP surface which may also give a definitive evidence for mechanism of synthesis of AuNPs via Turkevich method. Thus, for understanding the quantification of citrate ions in alkaline condition, the pH of the purified Au-18 after re-dispersion in water was adjusted to 11.3 using 0.1 M sodium hydroxide in order to reduce citrate layers formed by hydrogen bonding between adsorbed citrate and free citrate^21^.

**Figure 3:**
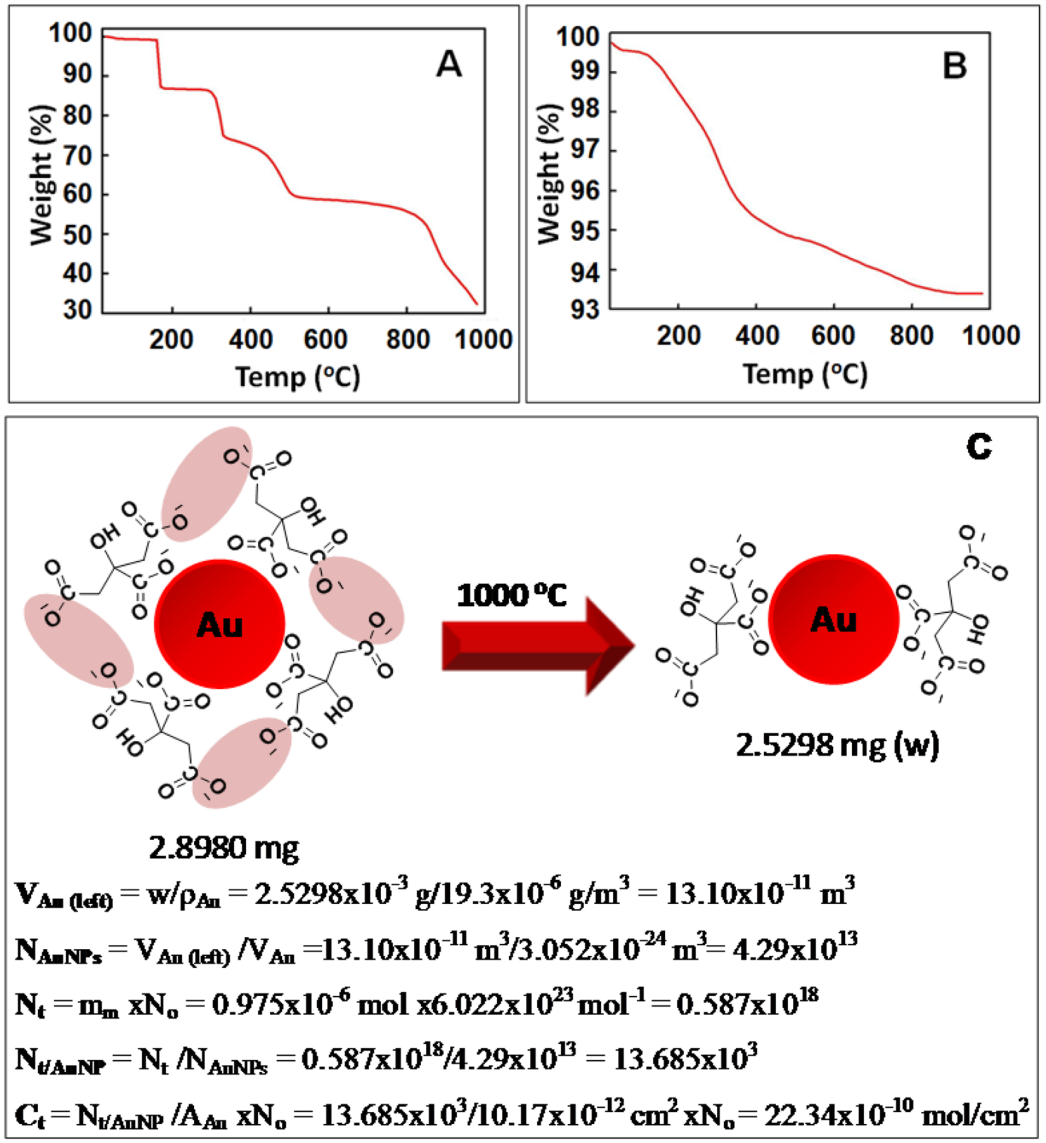
TG curve (Batch I) (**A**) MSC, (**B**) Au-18 at pH 11.3; and (**C**) Schematic reaction showing decomposition of Au-18 (at pH 11.3).

The decomposition behaviour of Au-18 (at pH 11.3) and TSC was compared using TGA (Fig. 3A,B). However, in contrast to the decomposition behaviour of MSC which completely decomposed, the TSC does not decompose entirely at 1000 °C as 70% mass decomposition was observed (Fig. 3A)^23^. On similar lines, when Au-18 at pH 11.3 (2.8980 mg) were heated up to same temperature, 93% of mass remained (2.5298 mg) which presumably contain 30% residual mass of TSC (Fig. 3B). This residual mass of TSC was accounted for calculation of citrate ion per AuNP and concentration of citrate ions per unit area at pH 11.3 as per previously discussed Eqs. 1–5. From the calculations, N_t/AuNP_ and C_t_ was calculated as 13.685 × 10^3^ and 22.34 × 10^−10^ mol/cm^2^. This experiment was performed in triplicates (Batch II and Batch III) for Au-18 (Figs. S3 and S4) and the average N_t/AuNP_ was determined as 5.5 ± 0.4 at pH 6.7 and 13.0 ± 2.2 at pH 11.3 respectively, further the average C_t_ was deduced as 9.0 ± 0.6 mol/cm^2^ at pH 6.7 and 21.2 ± 3.6 mol/cm^2^ at pH 11.3. The complete calculations deduced from all three measurements have been summarized in Table S1.

Thus, it can be clearly seen that the number of citrate ion found at pH 11.3 (13.0 ± 2.2 × 10^3^) were much higher than that at pH 6.7 (5.5 ± 0.4 × 10^3^). Interestingly, contrary to general belief and previous report^21^, the surface density of TSC on AuNP surface was observed to be much higher under alkaline conditions. Although the TSC ions are unable to form hydrogen bonds as MSC ions which is probably the reason for earlier reporting, the explanation for increased concentration TSC ions under basic condition is the alternative stronger ionic interactions between sodium salts of carboxylate anions. In other words, the enhancement of negatively charged carboxylate anions of citrate at higher pH results in the increase of citrate ions under alkaline conditions.

**Figure 4:**
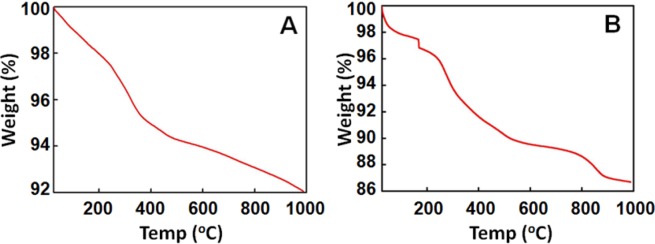
TG curve (Batch I) (**A**) Au-15 at pH 6.7 and (**B**) Au-15 at pH 11.3.

To verify the applicability of present method for assessment of the quantification of citrate ions for different sizes of AuNPs, Au-15 nanoparticles were analyzed in similar manner as described earlier at pH 6.7 and 11.3 using TGA (Fig. 4A,B).

Analogous to the calculation template discussed for Au-18 nanoparticles, N_t/AuNP_ for Au-15 was calculated to be 7.176 × 10^3^ at pH 6.7 (Fig. S5) and 18.276 × 10^3^ at pH 11.3 (Fig. S6). Correspondingly, C_t_ was found to be 16.87 × 10^−10^ mol/cm^2^ at pH 6.7 (Fig. S5) and 42.98 × 10^−10^ mol/cm^2^ at pH 11.3 (Fig. S6) respectively. This experiment was also performed in triplicates (Batch II and Batch III) for Au-15 (Figs. S7 and S8) and the average N_t/AuNP_ was determined as 7.3 ± 1.0 at pH 6.7 and 16.6 ± 1.6 at pH 11.3 respectively, further the average C_t_ was deduced as 17.2 ± 2.5 mol/cm^2^ at pH 6.7 and 39.0 ± 3.7 mol/cm^2^ at pH 11.3. The complete calculations deduced from all three measurements have been summarized in Table S2.

The data for Au-15 correspond to that of Au-18 in a way that N_t/AuNP_ along with C_t_ are augmenting as pH is increased (contrary to earlier hypothesis)^21^. It leads to another interesting observation that change in concentration of TSC does not have significant effect on the quantification trend of citrate ion on AuNPs at different reaction conditions. Importantly, these figures validate the TGA method for quantitative analysis of citrate ions on the surface of gold as well as give important inputs about the behaviour of citrate ions at neutral and basic pH.

**Figure 5:**
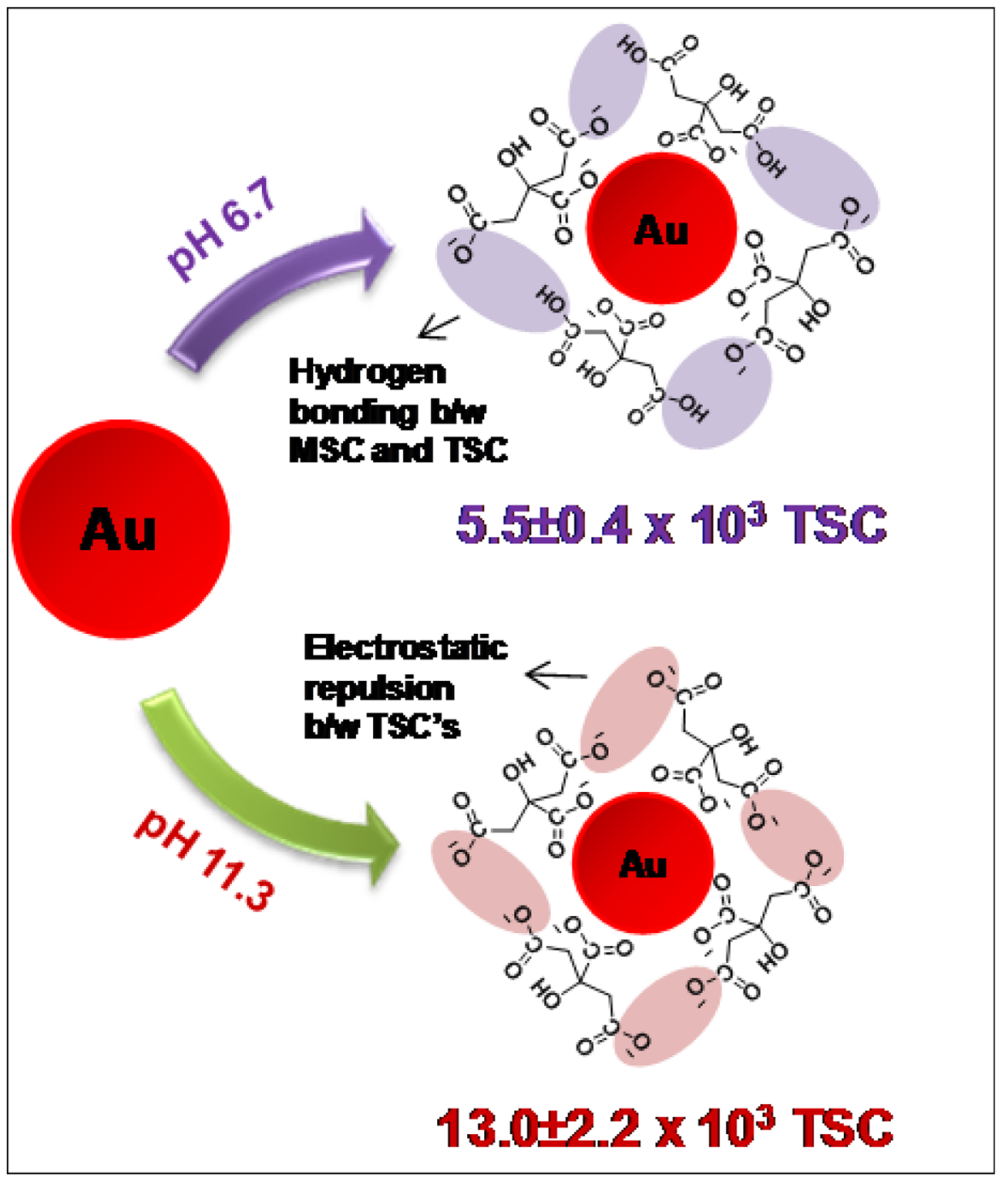
Graphical representation illustrating pH dependent quantification of citrate ions on AuNP surface.

In summary, TGA has been demonstrated as a new tool for the quantitative determination of citrate anions present on the AuNP surface. In earlier reports, mechanism of AuNPs synthesis was discussed only from the point of qualitative aspect but not quantitative. Thus, the determination of actual number of citrate ions present on the surface of AuNPs is essential as well as crucial for real understanding of the structure of AuNPs synthesized by Turkevich method. Also the understanding of the quantification on the surface of nanomaterials is crucial for mechanistic study of their synthetic pathway. For this purpose, two different sized AuNPs were synthesized by varying the concentration of TSC and were subjected to TGA analysis at different reaction conditions by varying the pH of the colloidal solutions. Based on the decomposition behaviour of thermogravimetric curve, it was observed that the number of citrate ions present on the surface of nanoparticle is highly pH dependent and was relatively much more in alkaline conditions which contradict the earlier hypothesis (Fig. 5). This was explained on the basis of the stronger ionic interactions between sodium salts of carboxylate anions which were observed under basic conditions. It is pertinent to mention here that this is the first report related where quantification of citrate ions present on AuNPs was done. Also, the change in the concentration of TSC does not make any significant effect on the trend of quantification of citrate ions. These experiments have been extensively characterized and validated to fully understand the adsorption of citrate ions.

The out comings of this work set up a platform to study the surface of nanoparticle and provide new methodology to quantify the number of ligand/biomolecule present on the nanoparticle surface which was not possible earlier. It resolves the level of understanding of the mechanism of formation of AuNPs. These findings also underline the importance of using TGA as a new tool for quantification of citrate anions on gold nanoparticle surface. It provides new heights of understanding to very popular Turkevich method. This detailed study will provide new insight in the up growing field of nanotechnology. Further research to apply this methodology on other materials is underway.

## Methods

### Reagents

Tetrachloroauric acid (HAuCl_4_), tri-sodium citrate (TSC), mono-sodium citrate (MSC) and sodium hydroxide were obtained from Sigma, India and were used as received. Ultrapure water used in the synthesis has a resistivity of 18 MΩ. All the glassware and magnetic beads used in the experiment were cleaned using aqua regia and rinsed with ultrapure water.

### Synthesis and characterization of gold nanoparticles

#### Synthesis of AuNPs

To the boiling solution of 0.25 mM, tetrachloroauric acid; varying concentrations (34 mM, 68 mM) of an aqueous solution of TSC was added. The heating was continued until a deep ruby red color appeared. The particles were purified using repeated cycles of centrifugation at 9000 rpm and re-dispersing in ultrapure water. The resulting particles were characterized using UV-visible (UV-vis) spectroscopy and transmission electron microscopy (TEM). The process was scaled up by taking a much larger volume (500 ml or more) of tetrachloroauric solution so that more than 4 mg of dry AuNPs could be isolated. The particles were prepared at two pH values (pH 6.7 & pH 11.3) and their pellet was lypholized for thermogravimetric analysis (TGA).

### Characterization of AuNPs

#### UV-visible spectroscopy

UV-visible spectrophotometer (Jasco, V-530) was used to confirm the formation of AuNPs by checking their surface plasmon resonance.

### Transmission electron microscopy (TEM) analysis

TEM technique was used to determine the shape and size of the AuNPs. Sample was prepared on carbon coated grid and dried before analysis under TEM (H-7500, Hitachi) operating at an acceleration voltage of 40 kv to 120 kv equipped with CCD camera with a resolution of 0.36 nm.

### Thermogravimetric analysis (TGA)

TGA-SDT Q 600, TA was used in the temperature range of 25-1000 °C heating at the rate of 20° per minute. The solid samples of MSC and TSC (as purchased) and AuNPs (after lyophilisation) were analyzed using TGA.

## Supplementary information

Supplementary Information.
