# Supplementary material for: Quantification of adsorbed and dangling citrate ions on gold nanoparticle surface using thermogravimetric analysis
Source: Sci Rep. 2020 May 19;10:8213. doi: 10.1038/s41598-020-65013-0 (PMC7237423; doi:10.1038/s41598-020-65013-0)
Supplement: Supplementary file 1 — Supplementary Information. [file 41598_2020_65013_MOESM1_ESM.docx]

**Quantification of adsorbed and dangling citrate ions on gold nanoparticle surface using thermogravimetric analysis**

Manish Bajaj,^1^ NishimaWangoo,^2^ D. V. S. Jain^1^ and Rohit K. Sharma^1,^*

^1^Department of Chemistry and Centre for Advanced Studies in Chemistry, Panjab University, Chandigarh-160014, India

^2^Department of Applied Sciences, University Institute of Engineering and Technology, Panjab University, Sector 25, Chandigarh-160014, India

**Corresponding Author**

***[rohitksg@pu.ac.in](mailto:rohitksg@pu.ac.in)

**Figure S1.** UV-vis spectra of Au-18 and Au-15.

**Figure S2.** TEM image (A) Au-18 and (B) Au-15 and their respective histogram size distributions are shown in (C) Au-18 (D) Au-15.

**Figure S3.** TG curve (Batch II) (A) Au-18 at pH 6.7 and (B) Au-18 at pH 11.3.

**Figure S4.** TG curve (Batch III) (A) Au-18 at pH 6.7 and (B) Au-18 at pH 11.3.

**Table S1. Quantification of citrate molecules on Au-18.**

|  | pH 6.7 | | pH 11.3 | |
| --- | --- | --- | --- | --- |
| Batch | ^a^C_n_/AuNP  (x 10^3^) | ^b^C_c_/AuNP  (x 10^-10^) | ^a^C_n_/AuNP  (x 10^3^) | ^b^C_c_/AuNP  (x 10^-10^) |
| I | 5.6 | 9.1 | 13.7 | 22.3 |
| II | 5.8 | 9.5 | 10.6 | 17.2 |
| III | 5.1 | 8.3 | 14.8 | 24.2 |
| Average | 5.5 ± 0.4 | 9.0 ± 0.6 | 13.0 ± 2.2 | 21.2 ± 3.6 |
| ^a^C_n_: Number of citrate molecules per AuNP; ^b^C_c_: Concentration of citrate molecules per AuNP (mol/cm^2^) | | | | |

**Figure S5.** Calculation of citrate molecules (Batch I) on the surface of Au-15 (at pH 6.7).

**Figure S6.** Calculation of citrate molecules (Batch I) on the surface of Au-15 (at pH 11.3).

**Figure S7.** TG curve (Batch II) (A) Au-15 at pH 6.7 and (B) Au-15 at pH 11.3.

**Figure S8.** TG curve (Batch III) (A) Au-15 at pH 6.7 and (B) Au-15 at pH 11.3.

**Table S2. Quantification of citrate molecules on Au-15.**

|  | pH 6.7 | | pH 11.3 | |
| --- | --- | --- | --- | --- |
| Batch | ^a^C_n_/AuNP  (x 10^3^) | ^b^C_c_/AuNP  (x 10^-10^) | ^a^C_n_/AuNP  (x 10^3^) | ^b^C_c_/AuNP  (x 10^-10^) |
| I | 7.2 | 16.9 | 18.3 | 43.0 |
| II | 6.3 | 14.9 | 15.2 | 35.7 |
| III | 8.4 | 19.9 | 16.3 | 38.4 |
| Average | 7.3 ± 1.0 | 17.2 ± 2.5 | 16.6 ± 1.6 | 39.0 ± 3.7 |
| ^a^C_n_: Number of citrate molecules per AuNP; ^b^C_c_: Concentration of citrate molecules per AuNP (mol/cm^2^) | | | | |
